# Promotions of Veterinarians in the Bureau of Animal Industry

**Published:** 1900-12

**Authors:** 


### PROMOTIONS OF VETERINARIANS IN THE BUREAU OF ANIMAL INDUSTRY.

The following are promotions of veterinarians in the Bureau of Animal Industry from September 1 to November 30, 1900 :

| | | | |
|---|---|---|---|
| Dr. A. M. Adams, Clarinda, Iowa, | from | $1200 to | $1400 |
| Dr. F. W. Ainsworth, Pittsburg, Pa., placed in charge of station, | " | " | " |
| Dr. Boyd Baldwin, Chicago, | " | " | " |
| Dr. A. E. Behnke, inspector in charge at Milwaukee, | " | 1400 to | 1600 |
| Dr. Tait Butler, transferred from Chicago and placed in charge at Cudahy, Wis., | " | 1200 to | 1400 |
| Dr. Thomas Castor, East Las Vegas, N. M., | " | " | " |
| Dr. H. A. Christmann, Washington, D. C., | " | " | " |
| Dr. W. Ross Cooper, Kansas City, | " | " | " |
| Dr. A. A. Holcombe, Chicago, promoted to travelling inspector, | " | " | " |
| Dr. Robert L. Kelly, East St. Louis, Ill., | " | " | " |
| Dr. Peter I. Kershner, Manhattan, Kan., | " | " | " |
| Dr. John A. Kiernan, Boston, | " | " | " |
| Dr. F. B. McCall, Chicago, | " | " | " |
| Dr. John P. O'Leary, Buffalo, | " | " | " |
| Dr. Harry D. Paxson, Fort Worth, Texas, | " | " | " |
| Dr. George W. Pope, Superintendent of Animal Quarantine Station at Garfield, N. J., | " | 1400 to | 1600 |
| Dr. Frank S. Tufts, Chicago, | " | 1200 to | 1400 |
| Dr. Harry N. Waller, formerly inspector in charge at Pittsburg, transferred to the same position at New York, and promoted | " | 1600 to | 1800 |

Dr. T. A. Geddes, stationed in England as inspector of livestock for importation.

Dr. R. W. Hickman, formerly inspector in charge at New York, transferred to Washington and placed in charge of the Miscellaneous Division of the Bureau.

